# Curcumin Nanocapsules Prepared With Native Casein Micelles Exhibit Potential to Reduce Paracetamol‐Induced Oxidative Stress

**DOI:** 10.1002/fsn3.70648

**Published:** 2025-09-26

**Authors:** Ankita Hooda, Bimlesh Mann, Abhishek Dutt Tripathi, Nandita Das, Himanshu Kumar Rai, Sulaxana Singh, Javed Masood Khan, Aparna Agarwal, Dipendra Kumar Mahato

**Affiliations:** ^1^ Department of Dairy Science and Food Technology Bananas Hindu University Varanasi India; ^2^ EP and HS, Indian Council of Agricultural Research, ICAR New Delhi India; ^3^ Food Safety and Standards Authority of India New Delhi India; ^4^ Department of Dairy Science and Food Technology Institute of Agricultural Sciences, Banaras Hindu University Varanasi India; ^5^ Food Inspection Organization, Ministry of Defense, Government of India New Delhi India; ^6^ Department of Food Science and Nutrition King Saud University Riyadh Saudi Arabia; ^7^ Department of Food Technology Lady Irwin College, University of Delhi New Delhi India; ^8^ CASS Food Research Centre School of Exercise and Nutrition Sciences, Deakin University Burwood Victoria Australia

**Keywords:** antioxidant, antioxidant indices, curcumin, nanoencapsulation, oxidative biomarkers, paracetamol

## Abstract

The study was designed to validate the antioxidant effect of curcumin nano encapsulated (NE) inside native Casein (CN) micelles using a small animal model. Paracetamol (P) is a regularly used drug that is safe when used at therapeutic levels, but an overdose of it has the potential to cause nephron and hepato‐toxicity. Thereby, the effect of curcumin, which has anti‐oxidative properties, was tested on the hepato‐nephron toxicity caused by the overdose. This would prove the effective protection offered to curcumin, by native CN micelles. Male albino mice were treated with nano capsules for 4 days (orally at 30 mg/kg of BW per day) before and after orally administering P (300 mg/kg of BW) for 2 days. There were six groups, including preventive and curative (NE and pristine curcumin) and two control groups. The negative control mice were administered water only for 6 days, and the other group was administered P at 300 mg/kg of BW, but no nano capsules/curcumin were administered. BUN levels were observed to increase in NE powder treated groups in comparison to the P group. The creatinine, glutamate pyruvate transaminase, and alkaline phosphatase levels reduced in mice treated with NE powder after/before P treatment. The levels of antioxidant indices like catalase, glutathione peroxidase, superoxide dismutase, and lipid peroxidation were noted in liver homogenate. The mice group treated with nano‐capsules orally showed increased activities of enzymes and a decrease in thio‐barbituric acid reactive substances as compared to the P group. Hence, nano capsules can act as a potential antioxidant that delivers curcumin with good bioavailability.

## Graphical abstract

Ameliorative potential of nano capsules of curcumin was evaluated in an animal model. Paracetamol overdose produced oxidative stress in the small animals. The increase in the level of blood serum biomarkers like creatinine, ALP, and GPT level was compensated by feeding nano capsules in curative and preventive formats. BUN levels increased on feeding nano capsules. Antioxidant enzymes like catalase, GSPHx, and SOD increased, and TBARS levels reduced on feeding nano capsules. The nano capsules delivered the curcumin without degradation.

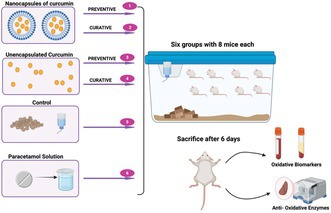

**Abbreviations**
ALP: Alkaline Phosphatase
BUN: Blood Urea Nitrogen
C: Control group
CAT: Catalase
CC: Curcumin curative group
CN: Casein
CP: Curcumin Preventive group
GPT: Glutamate Pyruvate Transaminase
GPx: Glutathione peroxidase
KAU: King‐ Armstrong Unit
MDA: Malondialdehyde
NC: Nano encapsulated Curative group
NE: Nano encapsulated
NP: Nano encapsulated Preventive group
P: Paracetamol group
SOD: Superoxide Dismutase
TBARS: Thio‐barbituric acid reactive substances

## 1 Introduction

Metabolic requirement causes constant generation of free radicals of different forms which then are effectively quenched by the antioxidant network of the body. Oxidative stress, which is caused by the production of reactive oxygen species (ROS) is the core factor responsible for severe cardiovascular diseases. It is the imbalance between the production and compensation of ROS (Bhimaraj and Tang 2012). According to (Li et al. 2022), oxidative stress is caused by a disparity between the body's oxidative and antioxidant reactions. This mismatch results in an excessive production of oxygen free radicals, which in turn creates a peroxidative environment that damages cells by interfering with their ability to repair themselves. Furthermore, it is widely accepted that the presence of excessive free radicals in the human body is closely associated with the development of a number of disorders (Zhang et al. 2021). ROS are highly reactive chemically, and when their number is in excess of what is required, the structural and functional integrity of that cell is damaged. This can directly modify DNA, lipids, and proteins, leading to chain reactions that bring oxidative damage (Chan et al. 2006; Pang et al. 2007).

Biomarker means a biological characteristic that can be observed, measured, and evaluated as an indicator of biological, pathogenic, or pharmacologic to therapeutic intervention. In case the generated free radicals exceed the number of antioxidants, the tissues and biomolecules are damaged in the body, finally leading to degenerative diseases (Gutteridge 1993). Imbalance of oxidants and antioxidants, where oxidants increase, leads to injury on membrane lipids, nucleic acid, and proteins (Toyokuni 1999). Acetaminophen (paracetamol, N‐acetyl p‐ aminophenol; APAP) which is an antipyretic agent, and high doses of this can cause renal tubular disease. Paracetamol induces oxidative stress resulting in lipoperoxidation, protein thiol oxidation, mitochondrial endoplasmic reticulum injury, altered homeostasis, and irreversible DNA damage (Sies 1993). Paracetamol, when used at therapeutic levels, is safe, but overdose causes fetal hepatic and renal necrosis in humans as well as experimental animals (Proudpot and Wright, 1970). The toxicity leads to increased levels of blood serum biomarker enzymes, namely glutamate pyruvate transaminase (GPT), alkaline phosphatase (ALP) and oxidation products like creatinine and blood urea nitrogen (BUN).

The aim of this particular study was to evaluate the antioxidant effects of curcumin encapsulated inside native casein micelles of skim milk against paracetamol‐induced toxicity, which was evaluated by measuring the levels of GPT, ALP, creatinine, and BUN from blood serum, and antioxidant indices like catalase, glutathione peroxidase, superoxide dismutase, and lipid peroxidation in the liver homogenate of experimental mice.

## 2 Materials and Methods

### 2.1 Chemicals

The milk for designing nanocapsules was procured from Dairy Farm, ICAR‐ NDRI, Karnal. Curcumin was procured from Plant Lipids Pvt. Ltd. The paracetamol (ACRO‐SIN 500, POCI Medicine India Pvt. Ltd.) was obtained from a local medical store, Karnal. The analyses of markers in blood serum and enzymes of liver homogenate were done by a commercially available diagnostic kit by ERBA Diagnostic Manheim, Germany. All other chemicals used in this research were of analytical grade from Sigma‐Aldrich (India), Merck (India).

### 2.2 Preparation and Optimization of Curcumin Nanocapsules Using Native Casein Micelles

Curcumin nanocapsules were prepared using native casein micelles derived from buffalo skim milk, without using synthetic micelle‐forming agents. Since native micelles were used, no critical micelle concentration (CMC) was required or applicable.

**FIGURE 1:**
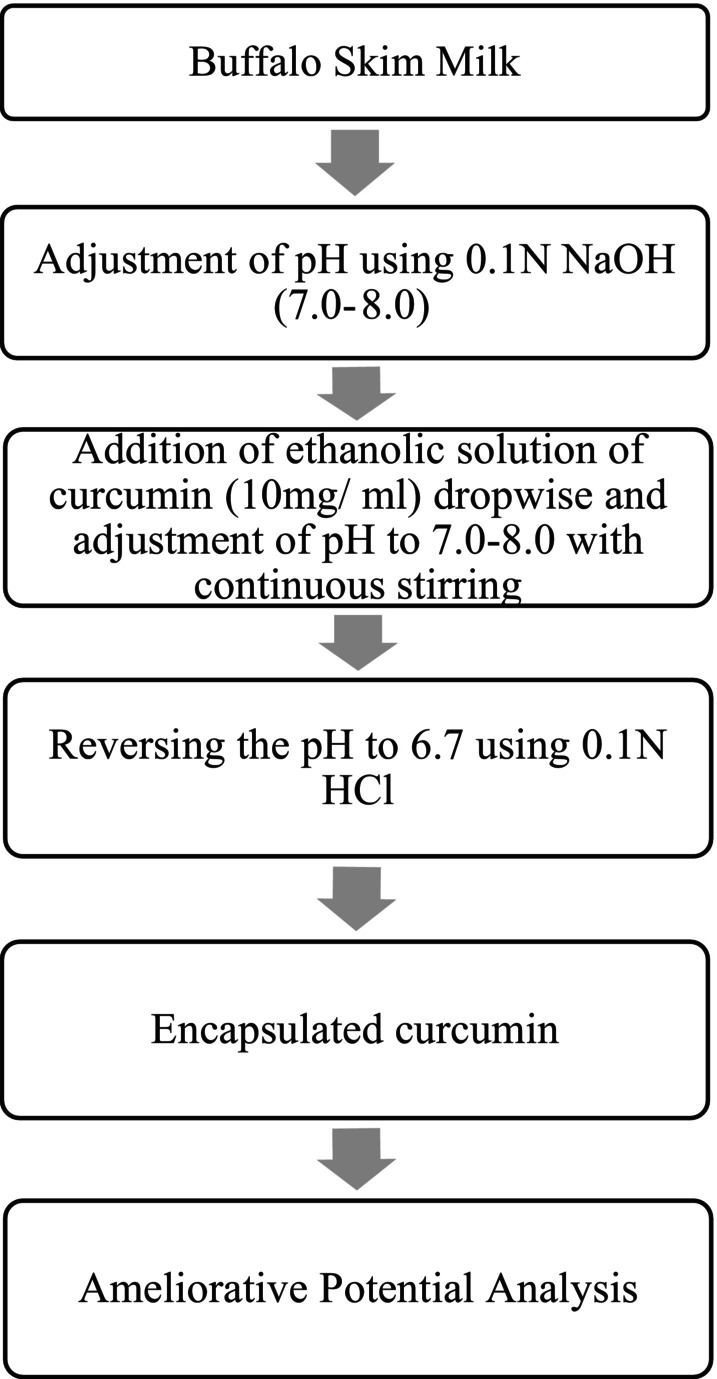
Preparation of Nanocapsules of Curcumin. The encapsulation was achieved through pH‐induced opening and closing of micelles in the presence of ethanol‐solubilized curcumin, as described by Hooda et al. (2025).

The method was based on the technique previously described by Hooda et al. (2025), wherein ethanol‐solubilized curcumin (10 mg/mL) was added dropwise to skim milk adjusted to pH 7.2 (Figure 1). This alkaline pH facilitated the temporary opening of the micellar structure, exposing hydrophobic binding sites. Continuous stirring ensured thorough interaction. The pH was then carefully returned to natural milk pH (6.7–6.8) to re‐establish the micelle structure with curcumin entrapped inside. The encapsulated solution was then concentrated and spray‐dried to obtain a powdered formulation.

This process preserved the native physicochemical and morphological properties of casein micelles, as confirmed by zeta potential, particle size analysis, TEM imaging, and encapsulation efficiency evaluation (Hooda et al. 2025).

### 2.3 Experimental Design and Animal Grouping

**FIGURE 2:**
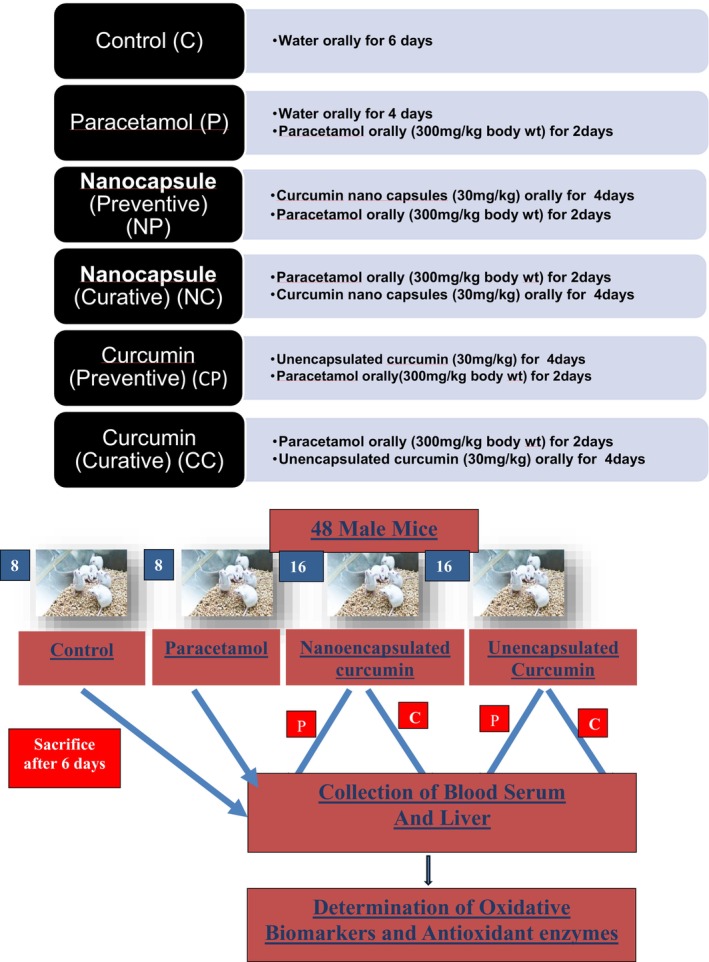
Feeding schedule for animal study.

To evaluate the ameliorative potential of curcumin nanocapsules against paracetamol‐induced oxidative stress, a controlled in vivo study was conducted using Swiss albino male mice (Figure 2).

- Species/Common Name: Swiss albino mice
- Gender: Male
- Age: 4 weeks
- Weight: 25–35 g
- Number of animals: 48 mice
- Housing: 8 mice per cage, maintained on a standard diet under a 12 h light/dark cycle at 22°C–24°C
- Ethical Approval: Approved by the Institutional Animal Ethics Committee (IAEC), ICAR‐NDRI, Karnal, India (Ref. No NDRI/IAEC/DC/155 dated 21‐11‐2018)

The animals were randomly divided into six groups (*n* = 8 per group). Each group received specific treatments orally as outlined below to simulate different phases of oxidative stress and intervention:

- Group 1 (Control): Received distilled water orally for 6 days.
- Group 2 (Paracetamol only): Received distilled water for 4 days, followed by paracetamol (300 mg/kg body weight) for 2 days to induce oxidative stress.
- Group 3 (Preventive NE curcumin—NP): Received curcumin nanocapsules (30 mg/kg BW) for 4 days, followed by paracetamol (300 mg/kg BW) for 2 days.
- Group 4 (Curative NE curcumin—NC): Received paracetamol (300 mg/kg BW) for 2 days, followed by curcumin nanocapsules (30 mg/kg BW) for 4 days.
- Group 5 (Preventive unencapsulated curcumin—CP): Received unencapsulated curcumin (30 mg/kg BW) for 4 days, followed by paracetamol (300 mg/kg BW) for 2 days.
- Group 6 (Curative unencapsulated curcumin—CC): Received paracetamol (300 mg/kg BW) for 2 days, followed by unencapsulated curcumin (30 mg/kg BW) for 4 days.

This study design allows for assessment of both preventive and curative effects of curcumin, in nanoencapsulated and unencapsulated forms, against paracetamol‐induced hepatic and renal oxidative stress.

### 2.4 Collection and Processing of Blood

The mice were sacrificed by exposing them to a jar of diethyl ether for a brief period of time. The blood was drawn by using a 1 mL syringe from the heart and collected in vials. These storage vials were kept at room temperature to allow it to clot and then centrifuged (3000 ***g*** for 10 min) in a centrifuge (Kubota, Japan) to separate serum to be further used for analysis of blood oxidative biomarkers.

### 2.5 Analysis of Blood Serum for Oxidative Biomarkers

The activity of all the markers and enzymes was analyzed as per instructions given in the kit. The markers and enzymes observed were Blood Urea Nitrogen (BUN), Creatinine, Alkaline Phosphatase (ALP), and Glutamate Pyruvate Transaminase (GPT). The kit for GPT was based on the measurement of dinitrophenyl hydrazine (2,4 DNPH) method of Reitman and Frankel (1957) (activity was expressed in international units per liter). Alkaline phosphatase (ALP) was based on the method by King and King (1954) which utilizes phenyl phosphate substrate (expressed as King and Armstrong Unit (KAU)). BUN was determined spectrophotometrically from serum samples using the Di‐acetyl‐monoxime (DAM) method (Crocker 1967; Marsh et al. 1965). Creatinine was measured by the method of Bonsnes and Taussky (1945). The measurements of both creatinine and BUN were expressed in milligrams per decilitre.

### 2.6 Collection of Liver and Preparation of Liver Homogenate

All the mice in each group were sacrificed and dissected, excising the entire liver. The liver was then washed using isotonic buffer (pH 7.4) and stored at −80°C (Athira et al. 2013). The liver was first weighed and then homogenized in the same. The homogenate was centrifuged to separate nuclei and cell debris according to the method of Athira et al. (2013).

### 2.7 Analysis of Liver Homogenate

The liver homogenate was analyzed for antioxidative enzymes viz. Catalase, Superoxide dismutase (SOD), Glutathione peroxidase (GSHPx) and lipid hydroperoxides. The CAT activity was estimated using instructions on the kit (units per milligram of protein). Superoxide dismutase was analyzed by the kit which utilized the inhibition of pyrogallol autooxidation by SOD. A single unit of enzyme is that amount which inhibits the reaction by 50% (units per milligram of protein) (Athira et al. 2013). Glutathione peroxidase was estimated using the kit (nanomoles of NADPH oxidized per minute per milligram of protein or unit per milligram of protein) spectrophotometrically. The detection of lipid peroxides (malondialdehyde) was done by the method of Suda et al. (1994) in terms of thiobarbituric acid reactive substances (TBARS) in liver homogenate samples (expressed as micromoles of malonaldehyde per milligram of protein).

### 2.8 Statistical Analysis

All statistical analyses were performed using Microsoft Excel and GraphPad Prism. Results are expressed as mean ± standard error of the mean (SEM) based on data from 8 animals per group.

To determine statistically significant differences among groups, one‐way Analysis of Variance (ANOVA) was employed for each biochemical and oxidative stress parameter. When ANOVA indicated a significant effect (*p* ≤ 0.05), Tukey's Honest Significant Difference (HSD) test was used as a post hoc test to identify specific group differences.

## 3 Results

### 3.1 Effect of Nano Encapsulated and Unencapsulated Curcumin on the Level of Biochemical Parameters in Blood Serum of Mice With Paracetamol Induced Hepato‐Nephrotoxicity

The level of ALP, GPT, and creatinine in blood serum was found to be increased for mice fed with an overdose of paracetamol (300 mg/kg body weight) due to hepato‐cellular necrosis and nephrotoxicity, which is a sign of liver damage (Table 1). High levels of GPT and ALP are signs of liver damage (Wang et al. 2012). Creatinine, which is a waste product of muscle tissues, at elevated levels, is indicative of renal diseases (Kashani et al. 2020). It must be noted that in the control group, mice were being fed a regular diet; in the P group, they were subjected to paracetamol overdose; nano‐encapsulated curcumin before and after the overdose was given in the NP and NC groups, and unencapsulated (UE) curcumin was given before and after the overdose in the CP and CC groups (Figure 4).

**TABLE 1:** Level of serum marker enzymes GPT, ALP, BUN, creatinine from blood serum of different mice groups.

Parameters/Groups	Control	Paracetamol	Nanoencapsulated curcumin		Unencapsulated curcumin	
			Preventive	Curative	Preventive	Curative
Blood Urea Nitrogen (mg/dL)	23.09 ± 0.10^e^	15.1 ± 0.34^a^	19.05 ± 0.14^b^	21.06 ± 0.06^c^	18.33 ± 0.12^d^	18.15 ± 0.03^d^
Creatinine (mg/dL)	0.40 ± 0.01^f^	1.18 ± 0.02^a^	0.70 ± 0.015^b^	0.49 ± 0.01^c^	0.89 ± 0.005^d^	1.14 ± 0.01^e^
GPT (IU/L)	30.62 ± 0.52^f^	101.29 ± 0.81^a^	45.96 ± 0.13^b^	40.13 ± 0.24^c^	81.03 ± 0.21^d^	70.63 ± 0.73^e^
ALP (KAU)	3.97 ± 0.12^b^	6.32 ± 0.19^a^	4.12 ± 0.08^b^	4.15 ± 0.15^b^	5.11 ± 0.10^c^	4.89 ± 0.09^c^

*Note:* Control: Normal control without any treatment, Paracetamol: Paracetamol treated mice at a dose of 300 mg/kg body weight for 2 days, Encapsulated curcumin: Nano encapsulated curcumin administration via skim milk powder (1) Preventive—Nano capsules of curcumin given at dose 30 mg/kg body weight for 4 days prior to paracetamol administration (2) Curative: Nano capsules of curcumin given at dose 30 mg/kg body weight for 4 days after paracetamol administration for 2 days, Unencapsulated curcumin—(1) Preventive –curcumin was given at a dose 30 mg/kg body weight for 4 days prior to paracetamol administration for 2 days (2) Curative—curcumin was given at a dose 30 mg/kg body weight for 4 days after paracetamol administration for 2 days: Values are expressed as Mean ± SEM for 8 mice per group. Values within the same row sharing different superscript letters differ significantly (*p* ≤ 0.05), based on one‐way ANOVA followed by Tukey's HSD post hoc test.

Feeding of skim milk powder with NE curcumin (30 mg/kg bw) before and after (preventive and curative) paracetamol overdose (300 mg/kg bw) lowered ALP, GPT, and creatinine levels significantly (*p* < 0.5). This level also decreased significantly (*p* ≤ 0.5) in blood serum of mice when fed with unencapsulated curcumin (30 mg/kg bw) after the overdose of paracetamol. The decrease was significantly less than the group fed with NE skim milk powder; i.e., more lowering effect was observed in the groups fed with nano encapsulated curcumin. GPT level increased significantly (*p* < 0.05) in the P group (101.29 ± 0.81 IU/L) from the C group (30.62 ± 0.52 IU/L). NE preventive group and NE curative group level reduced to 45.96 ± 0.13 and 40.13 ± 0.24 IU/L, the effect being slightly lower in the preventive group; i.e., to reduce the effects of the oxidative stress developed by paracetamol overdose, feeding curcumin after (or in curative mode) was more effective in comparison to giving it prior or in preventive mode. ALP levels also increased significantly in the P group (*p* < 0.05) which decreased towards control in NP and NC groups as well as CP and CC groups. Creatinine level increased significantly (*p* < 0.05) in the P group (66.10%). NP and NC group level reduced to 0.70 ± 0.01 and 0.49 ± 0.01 mg/dL; i.e., more towards control group (0.40 ± 0.01) i.e., towards the original values. The reduction in level is significant when mice were fed with UE curcumin, but significantly less when compared to NE group (*p* < 0.05). BUN (Blood Urea Nitrogen) level reduced significantly (*p* < 0.05) in the P group (34.6%). NP and NC group increased to 19.05 ± 0.14 and 21.06 ± 0.06 mg/dL; i.e., more towards control group (23.09 ± 0.10) (Figure 4, Table 1). The effect was observed to be slightly lower in the preventive group. The increase in level is significantly less for (*p* < 0.05) for mice group treated with UE curcumin than for NE curcumin.

The results obtained point out that the level of blood serum parameters in P, NP, NC, CP, and CC groups were disturbed due to an overdose of paracetamol when compared to the C group. When curcumin was fed to mice, the levels normalized toward the control group, but the normalization was more significant in the nano encapsulated (NP as well as NC) groups, as in the unencapsulated group, the curcumin may have been less bioavailable due to degradation in processing, storage, or digestion. In the nano encapsulated fed mice, the better effect was seen when the curative dose was given rather than the preventive dose.

### 3.2 Effect of Nano Encapsulated and Unencapsulated Curcumin on the Level of Antioxidative Enzymes in Liver Homogenate of Mice With Paracetamol Induced Hepato–Nephrotoxicity

**FIGURE 3:**
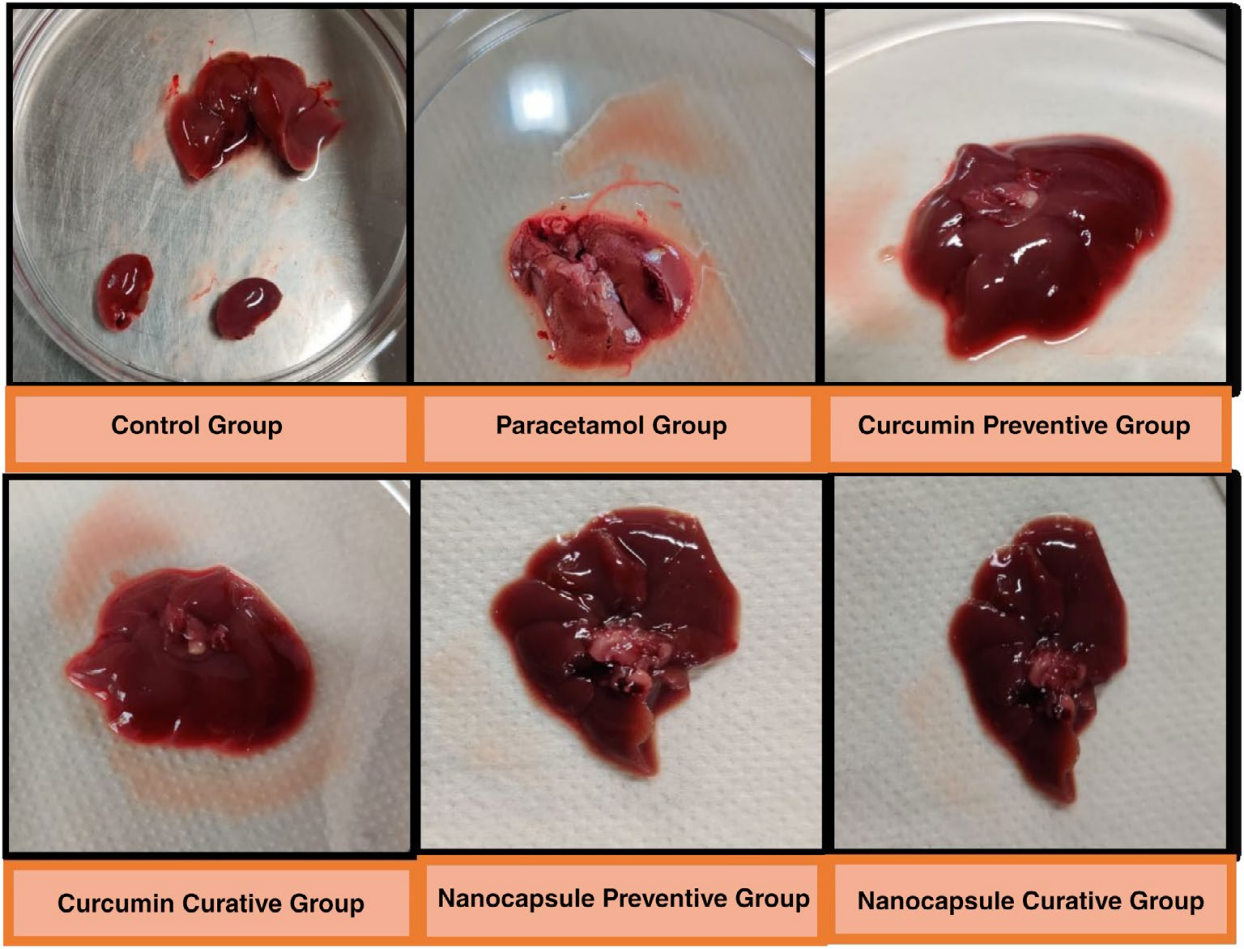
Liver images of various groups after 6 days of treatment of mice.

The activity of endogenous antioxidant enzymes in liver homogenate viz. catalase (CAT) superoxide dismutase (SOD), glutathione peroxidase (GSHPx), and radical species lipid hydroperoxide are depicted in Table 2. It was observed that the activity of CAT, SOD, and GSHPx in the paracetamol group was on the lower side as compared to the control group, that is, after an overdose of paracetamol. Similar to the blood serum parameters, the groups that were administered curcumin in encapsulated or unencapsulated form showed a significant (*p* ≤ 0.05) increase in the levels as compared to the paracetamol group, although the increase in the unencapsulated curcumin group was significantly less than the encapsulated groups (*p* ≤ 0.05) (Figure 3, Figure 5, Table 2).

**FIGURE 4:**
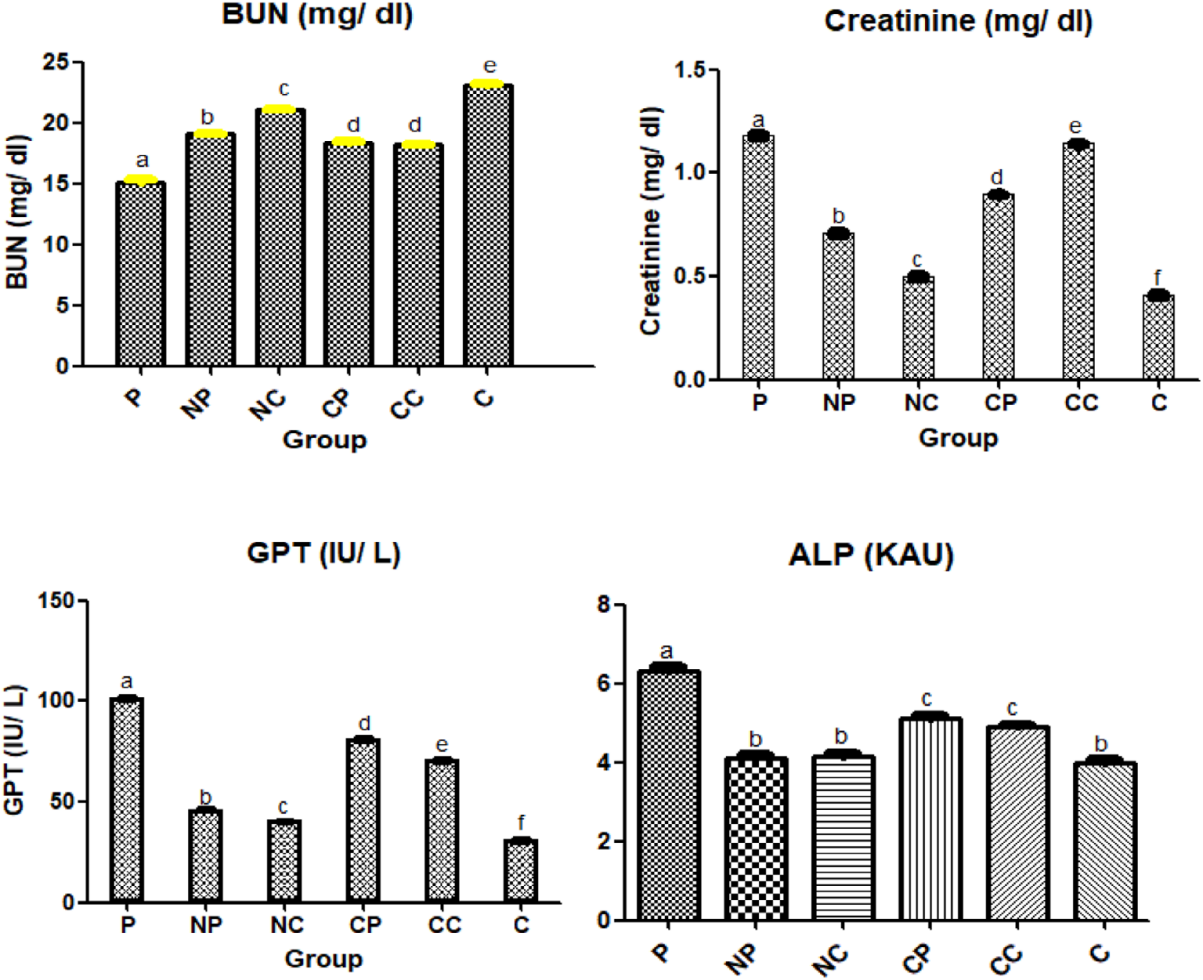
Level of oxidative biomarkers in blood serum of each group after 6 days of treatment. Control: Normal control without any treatment, Paracetamol: Paracetamol treated mice at a dose of 300 mg/kg body weight for 2 days, Encapsulated curcumin: Nano encapsulated curcumin administration via skim milk powder (1) Preventive—Nano capsules of curcumin given at dose 30 mg/kg body weight for 4 days prior to paracetamol administration (2) Curative: Nano capsules of curcumin given at dose 30 mg/kg body weight for 4 days after paracetamol administration for 2 days, Unencapsulated curcumin—(1) Preventive –curcumin was given at a dose 30 mg/kg body weight for 4 days prior to paracetamol administration for 2 days (2) Curative—curcumin was given at a dose 30 mg/kg body weight for 4 days after paracetamol administration for 2 days. Values are expressed as Mean ± SEM for 8 mice per group. Bars with different lowercase superscript letters indicate statistically significant differences between groups (*p* ≤ 0.05), as determined by one‐way ANOVA followed by Tukey's HSD post hoc test.

**TABLE 2:** Level of antioxidative enzymes CAT, SOD, Glutathione peroxidase, lipid peroxide in liver homogenate of different mice groups.

Parameters/Groups	Control	Paracetamol	Nanoencapsulated curcumin		Unencapsulated curcumin	
			Preventive	Curative	Preventive	Curative
Catalase*	23.09 ± 0.10^e^	15.1 ± 0.34^a^	19.05 ± 0.14^b^	21.06 ± 0.06^c^	18.33 ± 0.12^d^	18.15 ± 0.03^d^
SOD*	0.40 ± 0.01^f^	1.18 ± 0.02^a^	0.70 ± 0.015^b^	0.49 ± 0.01^c^	0.89 ± 0.005^d^	1.14 ± 0.01^e^
Glutathione Peroxide (GPx)*	30.62 ± 0.52^f^	101.29 ± 0.81^a^	45.96 ± 0.13^b^	40.13 ± 0.24^c^	81.03 ± 0.21^d^	70.63 ± 0.73^e^
Lipid Peroxide**	3.97 ± 0.12^b^	6.32 ± 0.19^a^	4.12 ± 0.08^b^	4.15 ± 0.15^b^	5.11 ± 0.10^c^	4.89 ± 0.09^c^

*Note:* Control: Normal control without any treatment, Paracetamol: Paracetamol treated mice at a dose of 300 mg/kg body weight for 2 days, Encapsulated curcumin: Nano encapsulated curcumin administration via skim milk powder (1) Preventive—Nano capsules of curcumin given at dose 30 mg/kg body weight for 4 days prior to paracetamol administration (2) Curative: Nano capsules of curcumin given at dose 30 mg/kg body weight for 4 days after paracetamol administration for 2 days, Unencapsulated curcumin—(1) Preventive –curcumin was given at a dose 30 mg/kg body weight for 4 days prior to paracetamol administration for 2 days (2) Curative—curcumin was given at a dose 30 mg/kg body weight for 4 days after paracetamol administration for 2 days: Values are expressed as Mean ± SEM for 8 mice per group. Values within the same row sharing different superscript letters differ significantly (*p* ≤ 0.05), based on one‐way ANOVA followed by Tukey's HSD post hoc test.

* U/mg protein.

** μMoles/Malondialdehyde/mg protein.

**FIGURE 5:**
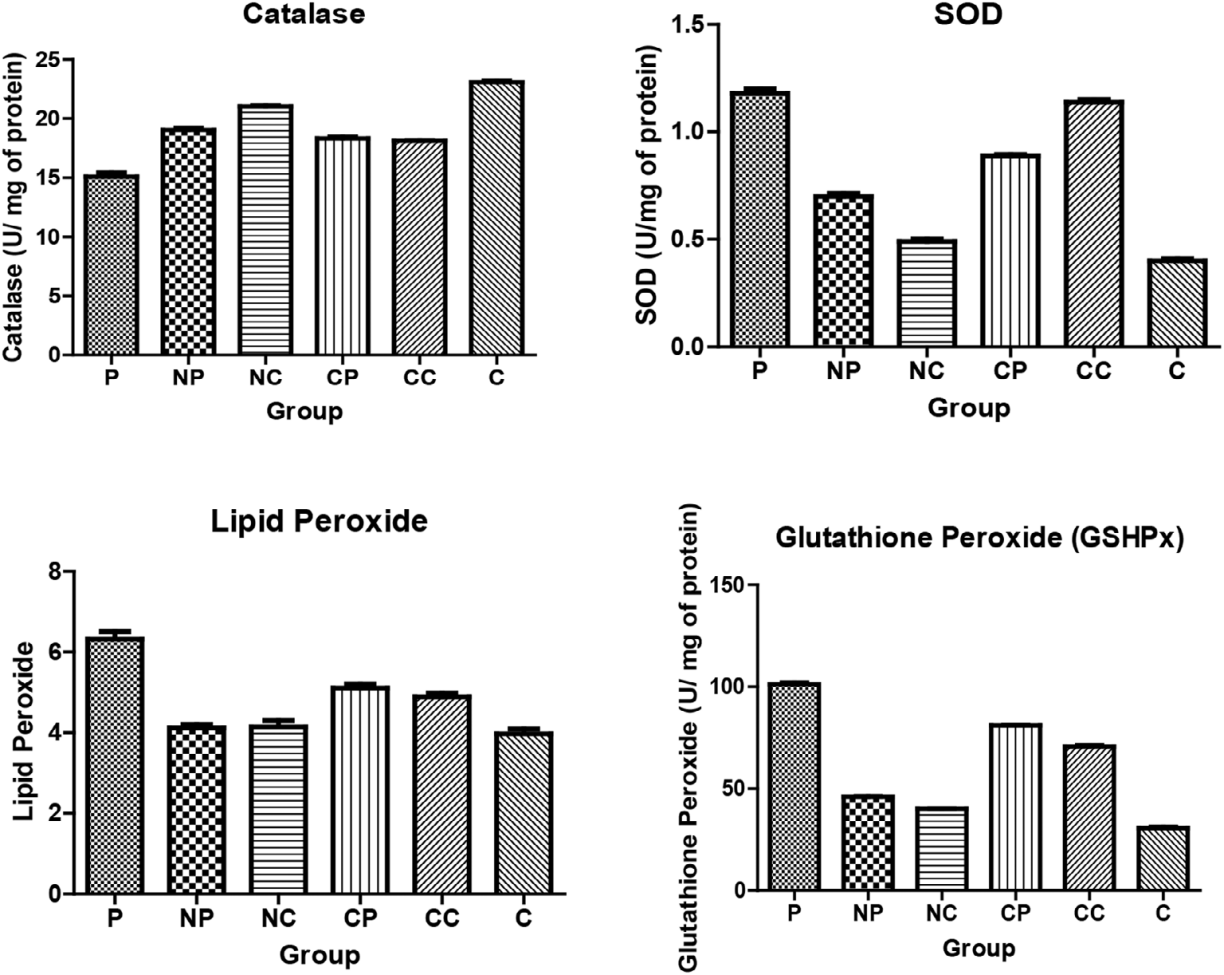
Level of oxidative biomarkers in blood serum of each group after 6 days of treatment. Control: Normal control without any treatment, Paracetamol: Paracetamol treated mice at a dose of 300 mg/kg body weight for 2 days, Encapsulated curcumin: Nano encapsulated curcumin administration via skim milk powder (1) Preventive—Nano capsules of curcumin given at a dose of 30 mg/kg body weight for 4 days prior to paracetamol administration (2) Curative: Nano capsules of curcumin given at a dose of 30 mg/kg body weight for 4 days after paracetamol administration for 2 days, Unencapsulated curcumin—(1) Preventive—curcumin was given at a dose of 30 mg/kg body weight for 4 days prior to paracetamol administration for 2 days (2) Curative—curcumin was given at a dose of 30 mg/kg body weight for 4 days after paracetamol administration for 2 days. Values are expressed as Mean ± SEM for 8 mice per group. Bars with different lowercase superscript letters indicate statistically significant differences between groups (*p* ≤ 0.05), as determined by one‐way ANOVA followed by Tukey's HSD post hoc test.

It was observed that the catalase activity increased significantly (*p* ≤ 0.05) in the mice group (NP and NC), that is, fed with NE and unencapsulated curcumin (Table 2). On the other hand, it was significantly lower in the paracetamol (150.24 ± 7.89 U/mg of protein) as compared to the control group (218.24 ± 8.11 U/mg of protein). The amount of increase was lower than other groups in the unencapsulated curcumin (preventive) mice group (165 ± 4.23 U/mg of protein). The value was, although significantly (*p* ≤ 0.05) more than the P group. SOD analysis showed that there is no significant difference (*p* > 0.05) in the level of enzyme activity in NP and NC groups (4.18 ± 0.22, 4.22 ± 0.32 U/mg of protein; respectively) when compared to the control and paracetamol groups. While NE curcumin (preventive and curative) significantly (*p* ≤ 0.05) increased the level of SOD towards normal value. Similarly, the GSHPx activity decreased significantly (*p* ≤ 0.05) in the P group (1.92 ± 0.12 U/mg of protein) as compared to the control group (3.12 ± 0.89 U/mg of protein). This effect is controlled by feeding NE curcumin as the level of GSHPx activity increased significantly (*p* ≤ 0.05) both in the preventive and curative group (2.9 ± 0.26 and 2.8 ± 0.21 U/mg of protein respectively). Unencapsulated curcumin dosage in group CC and CP (5 and 6) also showed a protective effect against paracetamol toxicity, but the effect is less significant (*p* ≤ 0.05) as compared to previous groups, as curcumin might have degraded (Figure 5, Table 2). The effect is not significantly different for NE preventive and curative groups. The thio‐barbituric acid reactive substance (TBARS) level is a indicative of lipid peroxidation and is generally expressed as increased malondialdehyde (MDA) formation. MDA values are indicative of oxidative lipid damage apart from being a product of the oxidation of polyunsaturated fatty acids. The level of TBARS increased significantly in the P group as compared to the control (27.1 ± 2.25 μMoles and 15.45 ± 2.21 μ Moles Malondialdehyde/mg of protein respectively). On administration of encapsulated curcumin, the level reduced significantly (*p* ≤ 0.05) (15.25 ± 0.85 and 15.10 ± 1.22 μ Moles Malondialdehyde/mg of protein for preventive and curative groups, respectively).

## 4 Discussion

Paracetamol is known to induce the production of a metabolite N‐acetyl‐p‐benzoquinonmine (NAPQI) (Dahlin et al. 1984). At therapeutic level doses, this metabolite is generally stabilized by glutathione (GSH) conjugation. This results in elimination by the kidney whereas at overdoses of paracetamol, the GSH is not able to detoxify NAPQI. The excess NAPQI causes liver damage associated with oxidative stress (García‐Román and Francés 2020) and hence, in our study, an increase in the level of Glutamate pyruvate Transaminase (GPT), Alkaline phosphatase (ALP) and creatinine is reported in mice groups fed with excess paracetamol.

BUN level if it is higher or lower than normal, indicates malfunctioning of kidneys and liver damage, respectively (Waring et al. 2008). Serum GPT, ALP, and creatinine were higher in paracetamol‐fed group than in control, indicating oxidative stress (“<ajol‐file‐journals_82_articles_43067_submission_proof_43067–973–38,687‐1‐10‐20,090,528 (1).pdf>”). This is also indicative of liver damage (Wang et al. 2012). Elevated creatinine level is found to be associated with various renal diseases (Yousef et al. 2010). Paracetamol overdose induced hepatitis (GPT) is prevented by curcumin through improvement of liver histopathology and decreasing oxidative stress (Somanawat et al. 2013). Curcumin has the potential to protect from paracetamol‐induced oxidative injury, impairment of kidney function, and liver damage (Yousef et al. 2010). Due to its antiapoptotic properties, ability to capture reactive oxygen species (ROS), and ability to reduce free radicals through the activation of erythroid‐2‐related nuclear transcription factor 2, curcumin has the ability to inhibit the increase in the enzymes AST and ALT in paracetamol‐induced liver injury. This results in an increase in catalase, Superoxide Dismutase (SOD), Glutathione S‐Transferase (GST), and Glutathione Peroxidase (GPx) production in the nucleus, which is then released into the cytosol. Pan et al. (2014) also found that curcumin incorporated in casein nanoparticles had improved anti‐inflammatory activity against HCT‐116 and BxPC3 cells compared to curcumin dispersed previously in normal solvent. Analysis of liver histology showed that curcumin nanoparticles were found to be more effective than curcumin at preventing liver damage caused by paracetamol, according to Roenigk scores. Hepatocyte membrane injury results in the release of Alanine and Aspartate aminotransferase (ALT and AST respectively) into the bloodstream. By encouraging hepatocyte regeneration and maintaining the integrity of cell membranes, curcumin nanoparticles given at the same dose and time were able to stop the release of hepatic intracellular enzymes into the blood. Since curcumin nanoparticles are more bioavailable and soluble than curcumin itself, they can enter cells more readily and have a stronger effect (Jebastin and Narayanasamy 2023). Pan et al. (2014) claimed that free curcumin did not show an inhibitory effect on both HCT‐116 and BxPC3 cells. The researchers concluded that there was improved cell‐mediated activity and the encapsulated sample led to enhanced cell proliferation. Penalva et al. (2015) performed pharmacokinetic analysis in Wistar mice to detect serum concentration profiles of folic acid solution after a single injection (dose 1 mg/kg). Animals treated with folic acid‐rich casein nanoparticles showed a greater increase in serum levels than animals that received a wet vitamin solution. The oral bioavailability of folic acid, when used as casein nanoparticles, was observed as 52%, which was about 50% higher than the conventional aqueous solution. Zhen et al. (2013) examined the antitumor effect of CDDP‐loaded nanoparticles on a model of intravenous hepatic H22 mice and found that the inhibition of tumor growth of CDDP‐loaded nanoparticles is 1.5 times higher compared to the free CDDP. Nanoparticles were able to penetrate the tumor and deliver CDDP to the tumor as this affects cells far from the vasculature.

It has been observed that treatment by paracetamol overdose causes a significant inhibitory effect on the activities of GST, GPx, SOD, and CAT in rat plasma, liver, kidney, brain, lung, heart, and testis (Yousef et al. 2010). This may be attributed to the activation of metabolism by paracetamol causing major toxicity which triggers a rapid loss of GSH. This also leads to lipid peroxidation rate increasing in both liver (Jaeschke et al. 2003) as well as kidney (Newton et al. 1986). NAPQI, which is a reactive metabolite of paracetamol, is reported to link covalently to free sulfhydryl groups of Glutathione (GSH) and causes subsequent oxidation (Lee et al. 2003). Other pathways for nephrotoxicity induced by paracetamol overdose include the production of metabolites that are derived hepatically from P‐GSH conjugates (Reshi et al. 2020). An increase in the level of TBARS is indicative of lipid oxidation and hence tissue damage induced by paracetamol overdose (Şener et al. 2003). Researchers have also stated that superoxide ion and hydrogen peroxide are released when paracetamol is activated metabolically in a CYP 450 (Cytochrome) system (Wang et al. 2017). It has also been reported that a large and wide variety of ROS produced can cause an imbalance in the glutathione redox state, which, in normal conditions, that is, with no paracetamol induced toxicity, is maintained by GSH‐depleting (GPx and GST) and GSH‐replenishing (GR) enzymes (Halliwell, 1996). Hence, the reduction in the activity of GSH concentration affects the GST and GPx activity causing an intense increase in lipid peroxidation (Czeczot et al. 2006). Hence, there is a fall in antioxidative capacity, which is also indicated by a fall in SOD and catalase as these are involved in ROS detoxification (Mladenović et al. 2009).

It can be concluded from the above values that the protective effect imparted by curcumin is more when it is nano‐encapsulated as compared to its unencapsulated form. In liver injury caused by paracetamol, curcumin can raise antioxidant enzyme levels while lowering lipid peroxidation and liver enzyme levels. Curcumin's ability to bind NAPQI and reduce free radicals provides protection against lipid peroxidation, as seen by the reduction of the free radical malondialdehyde (MDA) (Jebastin and Narayanasamy 2023).

Pandey et al. (2020) reported the presence of di hydro ferulic acid and ferulic acid in the bile after oral curcumin administration in rats. Although the degradation products of curcumin are less efficient as compared to curcumin in its biological activities, these may be associated with the beneficial activities in vivo after administration (Rahimi et al. 2016). Similarly the present study showed that nano encapsulated curcumin is more effective in mitigating paracetamol induced toxicity in mice as compared to unencapsulated curcumin which may be due to the degradation of curcumin in the latter and subsequent decrease in activity. It has been observed that the level of antioxidant enzymes SOD, catalase and GPx increased in the mice groups fed with encapsulated curcumin at a dose of 30 mg/kg bw as compared to the control. Similar results were reported by Somanawat et al. (2013), who studied the effects after feeding curcumin along with paracetamol. The increase in the antioxidant enzymes may be attributed to the antioxidant activity of curcumin. This also indicates that NE curcumin is required in less amount for the similar effect because nanoencapsulation increases the bioavailability of curcumin. Yousef et al. (2010) has also reported that the presence of curcumin with paracetamol successfully lead to the rise in TBARS as well as restored the activities of antioxidant enzymes. To enhance the curcumin absorption by oral administration, liposome‐encapsulated curcumin (LEC) was prepared by Jebastin and Narayanasamy (2023) from commercially available lecithin and found that encapsulation enhanced the gastro‐intestinal absorption of curcumin. Athira et al. (2013) and Kleekayai et al. (2020), evaluated antioxidant properties of the whey protein hydrolysates (WPH) against paracetamol induced oxidative stress and found that WPH administration can reduce paracetamol induced liver damage by elevating antioxidant enzyme level and reducing the oxidative biomarkers in the blood serum. Chen et al. (1981) observed an increase in the activity of SOD of peroxidized red blood cells derived from vitamin E deficient animals. SOD catalyzes the reaction where superoxide anions are converted to H_2_O_2_ in cytosol of cell and mitochondrial. Thereafter the degradation of H_2_O_2_ to H_2_Oand O_2_ is catalyzed by other two enzymes namely CAT and GPx. Glutathione reductase regenerates reduced glutathione from oxidized molecule which needs hydrogen to function effectively obtained from reduced GSH. Glutathione reductase maintains cellular bonds of GSH. Curcumin have been observed to scavenge oxygen free radicals like superoxide anions and hydroxyl radicals which initiate lipid peroxidation. It has been found that curcumin inhibits this in rat liver microsomes and brain homogenates. Curcumin has also been found to protect hemoglobin from oxidation induced by nitrates (Alhusaini et al. 2021). Curcumin protected liver against injury as well as fibrogenesis caused by CCl_4_ (Lee et al. 2016). Histopathological characteristics of liver was also improved which can be attributed to reducing activity of serum AST and ALT. As described previously that the level of SOD and catalase increase as these act as first line of defense against free radicals and the defense mechanism of cells against oxidative stress results in this phenomenon (El‐Gendy et al. 2010).

## 5 Conclusion

Native casein micelles effectively protected curcumin from degradation, as demonstrated by their ability to mitigate oxidative stress induced by paracetamol overdose in mouse model. The curcumin‐loaded nano capsules provided significant protection against paraceramol‐induced hepatic damage, evident in both preventive and curative interventions. The toxicity resulting from paracetamol overdose was alleviated through multiple pathways, including the reduction of oxidative biomarkers such as GPT, ALP, creatinine, and TBARS, along with the restoration of BUN levels to values comparable to control group. The nano capsules in both preventive and curative levels significantly increased the antioxidant potential of hepatocytes by restoring the activities of antioxidant enzymes SOD, CAT, and GSHPx. Further, similar nanocapsules could be designed for various bioactive compounds, and their bioactivity could be tested in animal models to analyze the protection offered by native casein micelles.

## Author Contributions

**Ankita Hooda:** conceptualization (lead), data curation (lead), formal analysis (lead), investigation (lead), methodology (lead), project administration (lead), writing – original draft (lead), writing – review and editing (lead). **Bimlesh Mann:** conceptualization (lead), data curation (equal), funding acquisition (lead), project administration (equal), resources (lead), supervision (lead), writing – review and editing (lead). **Abhishek Dutt Tripathi:** data curation (equal), writing – review and editing (equal). **Nandita Das:** data curation (equal), investigation (equal). **Himanshu Kumar Rai:** data curation (equal), investigation (equal), software (equal), writing – review and editing (equal). **Sulaxana Singh:** conceptualization (equal), data curation (equal), writing – review and editing (equal). **Javed Masood Khan:** formal analysis (equal), funding acquisition (equal), writing – review and editing (equal). **Aparna Agarwal:** data curation (equal), writing – review and editing (equal). **Dipendra Kumar Mahato:** writing – review and editing (equal), funding acquisition (equal).

## Ethics Statement

The present study was approved by the Institutional Animal Ethics Committee (IAEC), ICAR‐ NDRI, Karnal, India.

## Conflicts of Interest

The authors declare no conflicts of interest.

## Data Availability

Data will be made available on request.
